# Intraspecies comparison of *Streptomyces pratensis* genomes reveals high levels of recombination and gene conservation between strains of disparate geographic origin

**DOI:** 10.1186/1471-2164-15-970

**Published:** 2014-11-15

**Authors:** James R Doroghazi, Daniel H Buckley

**Affiliations:** Institute for Genomic Biology, University of Illinois at Urbana-Champaign, Urbana, USA; Department of Crop and Soil Sciences, Cornell University, Ithaca, USA

**Keywords:** *Streptomyces*, Comparative genomics, Bioprospecting, Homologous recombination, Genome evolution, Core genome, Pan-genome

## Abstract

**Background:**

*Streptomyces* are widespread bacteria that contribute to the terrestrial carbon cycle and produce the majority of clinically useful antibiotics. While interspecific genomic diversity has been investigated among *Streptomyces*, information is lacking on intraspecific genomic diversity. *Streptomyces pratensis* has high rates of homologous recombination but the impact of such gene exchange on genome evolution and the evolution of natural product gene clusters remains uncharacterized.

**Results:**

We report draft genome sequences of four *S. pratensis* strains and compare to the complete genome of *Streptomyces flavogriseus* IAF-45-CD (=ATCC 33331), a strain recently reclassified to *S. pratensis*. Despite disparate geographic origins, the genomes are highly similar with 85.9% of genes present in the core genome and conservation of all natural product gene clusters. Natural products include a novel combination of carbapenem and beta-lactamase inhibitor gene clusters. While high intraspecies recombination rates abolish the phylogenetic signal across the genome, intraspecies recombination is suppressed in two genomic regions. The first region is centered on an insertion/deletion polymorphism and the second on a hybrid NRPS-PKS gene. Finally, two gene families accounted for over 25% of the divergent genes in the core genome. The first includes homologs of *bldB* (required for spore development and antibiotic production) while the second includes homologs of an uncharacterized protein with a helix-turn-helix motif (*hpb*). Genes from these families co-occur with fifteen pairs spread across the genome. These genes have evidence for co-evolution of co-localized pairs, supporting previous assertions that these genes may function akin to a toxin-antitoxin system.

**Conclusions:**

*S. pratensis* genomes are highly similar with exceptional levels of recombination which erase phylogenetic signal among strains of the species. This species has a large core genome and variable terminal regions that are smaller than those found in interspecies comparisons. There is no geographic differentiation between these strains, but there is evidence for local linkage disequilibrium affecting two genomic regions. We have also shown further observational evidence that the DUF397-HTH (*bldB* and *hpb*) are a novel toxin-antitoxin pair.

## Background

*Streptomyces* are ubiquitous bacteria with many uncommon features and important industrial uses. They produce over half of the clinically useful antibiotics and a host of other bioactive, pharmaceutically relevant compounds [1]. The name *Streptomyces* means twisted fungus, reflecting the morphological and life cycle traits that these bacteria share with fungi. While *Streptomyces* taxonomy is notably problematic, multi-locus sequence analysis (MLSA) approaches are helping to resolve species boundaries in the genus [2–4]. For example, measurements of MLSA divergence in relation to DNA-DNA hybridization (DDH) values indicate that 0.7-0.8% divergence of MLSA loci roughly delineates species boundaries in *Streptomyces* [3, 5, 6]. Though asexual, *Streptomyces* are capable of genetic exchange within and between species [7], and there is evidence of widespread horizontal gene transfer within and between species of the genus [8]. The implications of horizontal gene transfer for genome evolution within *Streptomyces* remains poorly described.

*Streptomyces* genomes deviate from those of other bacteria in several ways. There can be multiple genomes per cellular compartment, though asexual spores have a single genome copy. Hyphae elongate at the tip and form septa that define cellular compartments at regular intervals [9, 10]. Roughly ten to twelve genomes can coexist inside of a single compartment [7, 11]. *Streptomyces* genomes are linear and replicate from a bidirectional central *ori*, although they can exist as unstable, circular molecules [12–14]. The ends of their chromosomes consist of terminal inverted repeats (TIRs), and the length of these TIR regions can vary largely, from 167 bp to 1 Mb [15, 16]. Their plasmids can be circular or linear, and can mobilize chromosomal markers at high frequency during interspecies transfer [17]. The chromosome has been classified into two sections: the central, conserved core region and the more variable terminal chromosome arms [18].

One surprise revealed by genome sequencing of *Streptomyces* species is the presence of numerous cryptic secondary metabolite gene clusters [19, 20]. These cryptic gene clusters encode products that are either silent or not identified as natural products during growth in the laboratory. For example, four decades of genetic analysis had identified four secondary metabolite gene clusters in the model organism *Streptomyces coelicolor*, but the first genome sequence revealed a total of 22 secondary metabolite gene clusters [19]. The diversity of natural product gene clusters is very high in *Streptomyces* compared to other bacterial genera. There is very little overlap in terms of shared natural product biosynthetic gene clusters between the currently closed *Streptomyces* genomes, all of which are from different species [21]. The intraspecies variability of natural product biosynthetic genes has not yet been determined through comparative genomic analysis of *Streptomyces* species. However, recent studies of *Salinispora*, another actinomycete genus rich in natural product biosynthetic genes, show high overall conservation within species [22].

The species *S. pratensis* has been described recently [23] to include isolates from a wide region of North America spanning sites found in North Carolina, New York, Michigan, and Quebec [8]. Nucleotide divergence of MLSA loci from strains of *S. pratensis* did not exceed 0.4%, justifying their inclusion in a single species [8]. Very high levels of homologous recombination were detected in *S. pratensis*, sufficient to promote linkage equilibrium for alleles at MLSA loci [8]. Interspecies recombination is widespread among *Streptomyces*, although interspecies gene exchange occurs at a much lower rate than intraspecies gene exchange [8]. The genetic coherence of *Streptomyces* species is surprising given the potential for widespread gene exchange, the high level of nucleotide similarity between many different species, and the null expectations for highly recombining populations [24, 25]. The maintenance of coherent genetic clusters that correspond to *Streptomyces* species suggests some mechanism for constraining interspecies gene exchange.

We have sequenced the genomes of four strains of the newly described species *S. pratensis*, including the type strain Ch24^T^ (=NRRL B-24916^T^). The four strains were isolated from edaphically similar sites separated by 740 km. These draft genome sequences were compared with the complete genome of *S. flavogriseus* IAF-45-CD (=ATCC 33331), which has been reassigned recently to *S. pratensis* [23].

## Methods

### Source of strains

*S. pratensis* strain IAF-45-CD (=ATCC 33331 = *S. flavogriseus* strain IAF-45-CD) was sourced directly from ATCC. *S. pratensis* IAF-45-CD was isolated from compost in Laval, Canada [26]. The other four strains of *S. pratensis* were isolated directly from soil. Strains Will23 and Will26 were both isolated from Willsboro, NY N 44.38, W -73.38. Strains Ch2 and Ch24^T^ = (NRRL B-24916^T^) were both isolated from Charlotte, NC (N 38.81, W -78.26), which is 740 km from the Willsboro site. Both sites are grassy fields which are edaphically similar. Isolation was carried out on glycerol-arginine media [27], including cycloheximide (300 mg L^-1^) and Rose Bengal (35 mg L^-1^) as described previously [28]. Classification of these strains as *S. pratensis* has been described previously.

### DNA preparation and sequencing

DNA was isolated as described previously [8]. DNA over 10 kb was selected using gel purification (MO-BIO, Carlsbad, CA) and submitted to the Cornell Life Sciences Core Laboratories Center for DNA sequencing. DNA libraries were prepared including MID tags and all four libraries were run together using GS FLX Titanium series reagents on a GS FLX instrument. Average read length for each isolate ranged from 422 to 425 bp. Additional summary genome statistics are given in Table 1. A *de novo* assembly was created with gsAssembler and a mapping assembly was created with gsMapper using the IAF-45-CD genome as reference. A final assembly was performed with gsAssembler using a combination of the original reads and faux reads created by breaking contigs from the independent gsAssembler and gsMapper runs into 1999 bp pieces with 50 bp overlap. Contigs were ordered using Mauve version 2.3.1 [29] and gene prediction was performed with Prodigal version 2.50 [30]. Genome sequences are available through Genbank BioProjects PRJNA252632-5.

**Table 1:** **Genome assembly summary statistics**

Strain	Assembled size (bp)	Contigs	N50 (bp)	ORFs	Reads	Coverage	G + C (%)
Will23	7517207 (7521185)	97 (112)	171112	6723	357319	20.0x	71.1
Will26	7510568 (7516455)	87 (110)	176481	6739	492343	27.9x	71.1
Ch2	7538829 (7544224)	128 (149)	128423	6782	284409	16.0x	71.1
Ch24	7623889 (7630757)	110 (137)	132873	6770	332240	18.5x	71.0
IAF-45-CD (pSFLA01, pSFLA02)	7337497 (188552, 130055)	1 (1, 1)	NA	6443 (201, 126)	NA	NA	71.1 (67.8, 67.2)

Data is provided both for large contigs (>2000 bp) and for all contigs (provided parenthetically). For IAF-45-CD, the two numbers in parentheses indicate data for the plasmids pSFLA01 and pSFLA02 respectively.

### Genome analyses

Mauve was used for genome alignment and to find positional orthologs and SNPs. The nucleotide sequences of core positional orthologs were aligned using ClustalW version 1.83 [31]. Distances were calculated with DNAdist in the PHYLIP package version 3.69 [32]. Annotation of divergent core genes was performed using Reverse Position Specific BLAST 2.2.25+ against the Conserved Domain Database (CDD.v2.32) [33, 34]. GO term enrichment was performed using topGO version 2.10.0 [35] within Bioconductor (Biobase version 2.18.0) [36]. Secondary metabolite biosynthetic gene clusters were found with AntiSMASH version 1.1.0 [37]. BldB and Hpb amino acid sequences were aligned using ClustalW version 1.83 with default alignment parameters [31]. The maximum likelihood trees were created using FastTreeMP version 2.1.5 [38]. Tree visualization was performed with the Python library ETE version 2.2 [39].

The other genomes used for the analyses of phylogenetic signal are: *Helicobacter pylori* F32 (NC_017366.1), *H. pylori* F57 (NC_017367.1), *H. pylori* F16 (NC_017368.1), *H. pylori* 51 (NC_017382.1), *H. pylori* F30 (NC_017365.1)*, Mycobacterium tuberculosis* str. Erdman = ATCC 35801 (AP012340.1), *M. tuberculosis* KZN 4207 (NC_016768.1), *M. tuberculosis* RGTB423 (NC_017528.1), *M. tuberculosis* CTRI-2 (NC_017524.1), and *M. tuberculosis* CCDC5079 (NC_017523.1). All analyses not described above were performed using custom Perl scripts. Significance testing for regions of extended linkage was performed using random draws to determine compatible sites based on the exponential decay discussed in the text across 20 kb tracts of the genome centered on every SNP and repeated 100 times.

## Results

### Genome summary statistics

The draft genomes for the *S. pratensis* strains (including plasmids) range from 7510568–7623889 bp (for contigs over 2000 bp) and have 6723 to 6782 predicted genes and an average G + C content of 71% (Table 1). The draft genomes do not allow for conclusive delineation between chromosomal and plasmid DNA due to the presence of linear plasmids. Increasing coverage (from 16× to 27×) corresponds to an increase in N50 from 128423 to 171112 and a decrease in the number of contigs (from 128 to 87) for each assembly; there is no relationship between coverage and assembled genome size.

### Conservation of gene content

**Figure 1:**
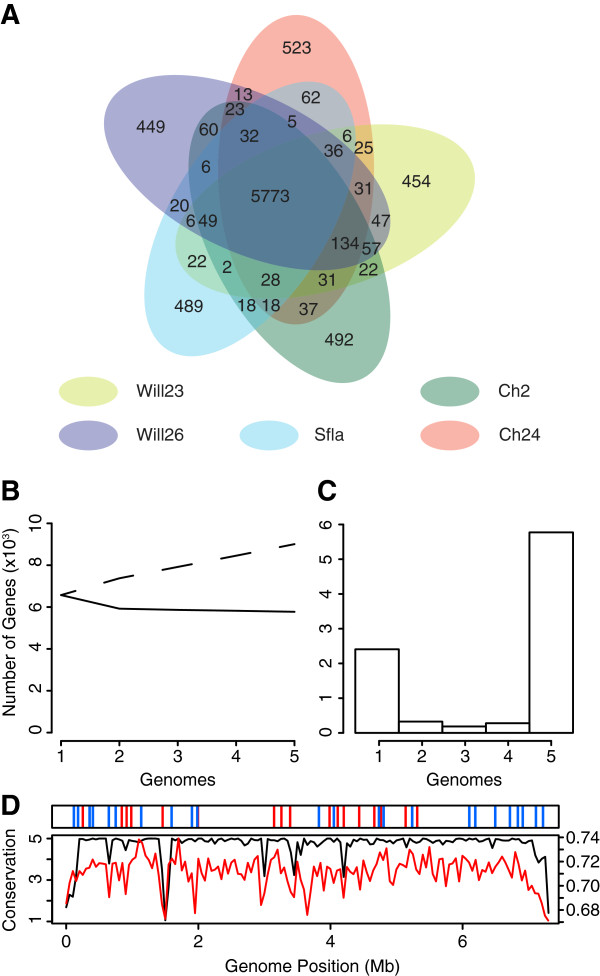
**Gene conservation in S.pratensis. (A)** Co-occurrence of positional orthologs across the five *S. pratensis* genomes. Note the low number of genes shared among two to four genomes. Most genes are either unique or conserved. **(B)** The size of the core (solid line) and pan (dashed line) genome in relation to numbers of genomes compared. **(C)** The number of unique positional orthologs that are shared by a given number of genomes. **(D)** The upper panel shows the locations of secondary metabolite gene clusters (blue bars) and the *bldB*/*hpb* genes (red bars). The lower panel shows the chromosome position of core and variable genes as the average number genomes sharing genes in a 50kb window (black line). Average genome G + C content (red line) is calculated for the same 50 kb windows.

To determine the similarity of gene content in the five genomes, we determined orthologous genes based on position and similarity in whole genome alignments calculated with Mauve. A surprising 5773 positional orthologs are shared by all five strains indicating that the core genome comprises 85-88% of gene content for the five genomes. Adding additional genomes beyond the second genome causes only a modest reduction in the size of the core genome (Figure 1B). There is a clear pattern that most genes are either unique to one strain (2,407 genes total, ranging from 449 to 523 unique genes per strain) or shared by all five strains (5773 genes) (Figures 1A and 1C). This pattern is established from all pairwise comparisons of genomes rather than comparison solely to IAF-45-CD, and thus is not likely to result from differences in coverage between complete and draft genomes. It is expected that the number of genes unique to one strain will be dependent on the total number of genomes sampled and hence the proportion of unique genes may decrease even further as more genomes of the species are sampled.

**Figure 2:**
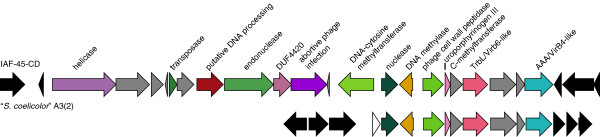
**A putative mobile element unique to IAF-45-CD.** A subset of genes from the large unique genomic island found at the 1.5 Mbp locus of IAF-45-CD. A region with identical gene content and high similarity (*e.g.* 99% predicted TrbL amino acid sequence identity) was found in the genome of “*S. coelicolor*” A3(2). For comparison, the *rpoB* and *gyrB* amino acid sequences of IAF-45-CD and A3(2) are 96% and 87% identical, respectively. Genes that have a putative functional assignment are shown in color, genes outside of the region of interest are shown in black, and genes with no putative functional assignment are shown in gray. Orthologous genes are shown in the same color.

Variable genes are not randomly distributed across the chromosome (Figure 1D). There are 11 genomic islands of 10 kbp or longer in IAF-45-CD that are enriched (≥50% of gene content) in genes specific to IAF-45-CD (Figure 1D). These islands account for 376505 bp of the genome, or 5.1% of the chromosome. The terminal regions of the chromosome are among the regions enriched in variable genes (Figure 1D), supporting the hypothesis that genome variability in *Streptomyces* increases at chromosomal termini. The terminal variable regions each comprise 112 kbp and 36 kbp of the chromosome, based on these criteria. Certain GO terms are overrepresented among the strain specific genes (Table 2 lists genes unique to IAF-45-CD). Primary among them are genes involved in transposition and integration, as well as metabolic processes. The largest variable region unique to IAF-45-CD is a putative mobile element likely to be involved in phage defense (Figure 2). This region, which lies between Sfla_1274 and Sfla_1300, contains multiple DNA methyltransferases, nucleases and a predicted gene for an abortive phage infection protein. This region also encodes putative transfer genes that are highly similar to a genomic neighborhood found in “*S. coelicolor*” A3(2) M145. Taken altogether, these results show that there is a highly similar overall coding capacity between these five intraspecific strains, and that the variability that does exist is isolated to chromosome termini and genomic islands likely to have been acquired through horizontal gene transfer.

**Table 2:** **GO term enrichment in genes unique to IAF-45-CD**

GO ID	Term	All	Unique	Expected	***p***-value
GO:0006259	DNA metabolic process	92	22	2.39	8.12E-14
GO:0015074	DNA integration	11	10	0.29	7.02E-13
GO:0003676	nucleic acid binding	439	34	11.29	3.40E-07
GO:0044260	cellular macromolecule metabolic process	700	42	18.2	8.12E-07
GO:0090304	nucleic acid metabolic process	555	37	14.43	1.09E-06
GO:0003677	DNA binding	356	29	9.16	3.47E-06
GO:0034641	cellular nitrogen compound metabolic pro…	699	41	18.18	3.47E-06
GO:0006310	DNA recombination	18	8	0.47	4.47E-06
GO:0006139	nucleobase-containing compound metabolic…	631	38	16.41	1.09E-05
GO:0006807	nitrogen compound metabolic process	725	41	18.85	1.19E-05
GO:0043170	macromolecule metabolic process	803	42	20.88	7.57E-05
GO:0006304	DNA modification	7	5	0.18	1.92E-04
GO:0004803	transposase activity	4	4	0.1	3.03E-04
GO:0006305	DNA alkylation	4	4	0.1	3.83E-04
GO:0006306	DNA methylation	4	4	0.1	3.83E-04
GO:0006313	transposition, DNA-mediated	4	4	0.1	3.83E-04
GO:0032196	transposition	4	4	0.1	3.83E-04
GO:0040029	regulation of gene expression, epigeneti…	4	4	0.1	3.83E-04
GO:0044237	cellular metabolic process	1096	48	28.5	1.09E-03
GO:0008152	metabolic process	2048	66	53.26	8.94E-03
GO:0043414	macromolecule methylation	7	4	0.18	1.28E-02
GO:0043412	macromolecule modification	63	9	1.64	2.28E-02
GO:0032259	methylation	16	5	0.42	3.28E-02

Gene ontology (GO) classifications were used to determine whether certain functional categories are enriched in the pool of genes unique to IAF-45-CD. The category ‘All’ indicates annotations for the entire IAF-45-CD chromosome; ‘Unique’ indicates annotations for genes unique to IAF-45-CD; ‘Expected’ indicates the number of unique genes expected if sampled at random from the pool of all unique genes found in the five genomes. The Bonferroni corrected *p*-values indicate the probability that the observed unique gene frequency is due to chance.

**Figure 3:**
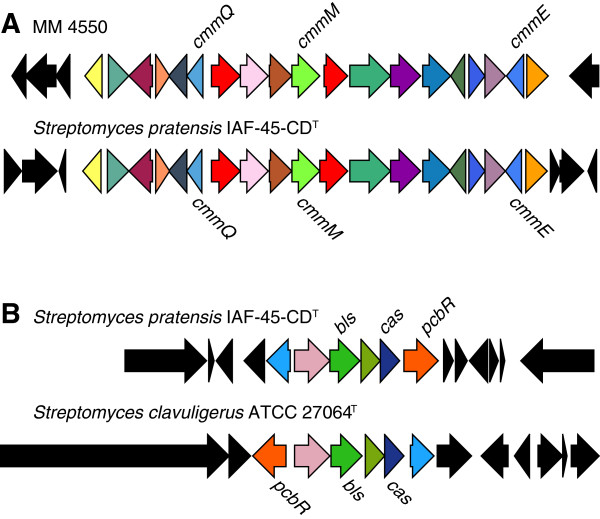
**Beta-lactam and beta-lactamase inhibitor biosynthetic gene clusters. (A)** The MM 4550 beta-lactamase inhibitor gene cluster from *S. argenteolus* is highly similar to a gene cluster found in *S. pratensis* IAF-45-CD (genes Sfla_0144-Sfla_0168). **(B)** The clavulanic acid gene cluster from *S. clavuligerus* (SCLAV_4187-SCLAV_4200) is highly similar to a gene cluster found in *S. pratensis* (genes Sfla_0550-Sfla_0565). Homologous genes are shown in the same color and genes outside of the similar regions are shown in black.

To determine the variation in natural product biosynthetic gene clusters between these five strains, we used positional orthologs as described above to determine the presence/absence of gene clusters detected using the program antiSMASH. All five strains have 100% conservation of putative secondary metabolic biosynthetic gene clusters in terms of presence (not sequence identity). *S. pratensis* contains a novel combination of biosynthetic gene clusters including one that synthesizes a beta-lactam antibiotic and another that synthesizes a beta-lactamase inhibitor (Figure 3). A similar combination of beta-lactam and beta-lactamase inhibitor gene clusters is present in *S. clavuligerus* ATCC 27064, and in fact the latter gene cluster in *S. pratensis* most closely resembles a gene cluster in *S. clavuligerus* ATCC 27064 which is responsible for production of the beta-lactamase inhibitor clavulanic acid. In contrast, the beta-lactam gene cluster in *S. clavuligerus* ATCC 27064 produces cephamycin and is distantly related to the beta-lactam gene cluster found in *S. pratentis*. The *S. pratensis* beta-lactam gene cluster most closely resembles that reported for MM 4550 from *S. argenteolus* ATCC 11009 [40]. MM 4550 is within the carbapenem class of beta-lactams [41], but is distantly related to the cephamycin gene cluster (e.g. the beta-lactam synthases from *S. clavuligerus* and *S. pratensis* share only 32% identity over 81% of *cmmM*). In addition, biosynthetic gene clusters in *Streptomyces* have been shown to associate only on short evolutionary time scales [42], suggesting that these two sets of gene clusters have different evolutionary origins *in S. pratensis* and *S clavuligerus*. Taken together, this shows that the pharmaceutically important association of these two antibiotic classes, a beta-lactam and a beta-lactamase (antibiotic resistance) inhibitor, has evolved independently on more than one occasion.

### Sequence level conservation

**Figure 4:**
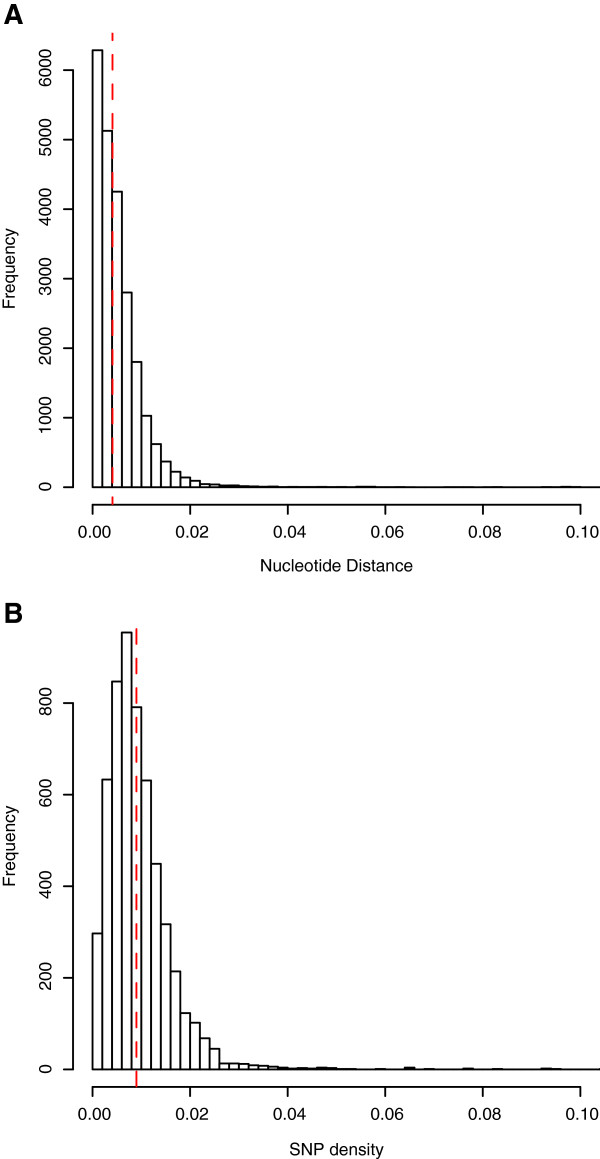
**Sequence conservation. (A)** Pairwise genetic distances in conserved genes between the four draft genomes and IAF-45-CD. Only genes conserved in all five genomes were used. The median pairwise distance, 0.0041, is shown with a dashed red line. **(B)** SNP density sampled in 50 kb windows across the genome. The median SNP density, 0.009, is shown with a dashed red line.

Genome-wide intraspecific sequence divergence was calculated from whole the genome alignment indicating a high level of sequence similarity. Core gene sequences from the four draft genomes had a median nucleotide dissimilarity to IAF-45-CD of 0.0041 (s.d. = 0.016, Figure 4A), or 99.59% nucleotide identity. The median density of SNPs calculated in 50 kb windows across the conserved regions of all five genomes is 0.009 (s.d. = 0.010, Figure 4B).

Core genes with more than 5% divergence to a positional ortholog in IAF-45-CD were identified and investigated. There are 59 such genes present in 33 regions across the genome (Table 3). These regions were delimited based on whether the identified genes co-occurred within 10 kbp. The 33 regions contained a median of 2 divergent genes and varied in length from 156 to 15570 bp. Two of these divergent regions of the core genome are worth noting. Sfla_4158 is predicted to encode a 51 amino acid lanthipeptide precursor in a putative two-component lanthipeptide biosynthetic gene cluster. The N-terminal 29 amino acids are identical across all *S. pratensis* genomes, but there are two indels and 11 point mutations resulting in six amino acid substitutions among the 22 amino acids at the C-terminal of Sfla_4158. As lanthipeptide precursors are post-translationally modified to form the final product, these amino acid changes must impact directly the final structure of this lanthipeptide. In the second region of interest are found Sfla_1620 and 1621 and 1627, which are annotated respectively as a peptidase, a hypothetical protein, and a gene required for antibiotic production and formation of aerial hyphae (*bldB*). Sfla_1620 is most similar to the type VII secretion-associated serine protease mycosin and Sfla_1621 is most similar (detected with DELTA-BLAST [43]) to ESX-1 secretion-associated alanine and proline rich proteins involved in the ESX-1 type VII secretion system in *Mycobacterium*. In *S. pratensis* these genes occur as part of an operon comprised of several genes that have orthologs required for morphogenetic development and spore formation in *“S. coelicolor”* A3(2) and which are regulated in part by BldB (Sfla_1627).

**Table 3:** **Highly divergent core genes**

Gene name	CD hit	Predicted function
Sfla_0019	N/A	hypothetical
Sfla_0256	pfam05120	Gas vesicle protein G
Sfla_0695	TIGR03062	Unknown, required for phage infection
Sfla_0790	pfam04149	BldB family
Sfla_0791	N/A	HBP
Sfla_0852	pfam04149	BldB family
Sfla_0853	N/A	HBP
Sfla_1620	TIGR03921	type VII secretion-associated serine protease mycosin
Sfla_1621	N/A	hypothetical
Sfla_1909	N/A	hypothetical
Sfla_1910	N/A	hypothetical
Sfla_1911	N/A	cholesterol esterase
Sfla_2255	N/A	hypothetical
Sfla_2258	N/A	hypothetical
Sfla_2259	N/A	hypothetical
Sfla_2260	N/A	hypothetical
Sfla_2267	N/A	TadE-family protein
Sfla_2678	pfam04149	BldB family
Sfla_2704	pfam07228	stage II sporulation E protein
Sfla_2785	smart00530	HBP
Sfla_2786	pfam04149	BldB family
Sfla_2892	COG0642	Signal transduction histidine kinase
Sfla_2916	pfam06259	alpha/beta hydrolase fold
Sfla_2921	N/A	HBP
Sfla_2922	pfam04149	BldB family
Sfla_2930	N/A	hypothetical
Sfla_2931	N/A	hypothetical
Sfla_3203	TIGR02614	FtsW - Biosynthesis and degradation of murein sacculus and peptidoglycan
Sfla_3555	pfam04149	BldB family
Sfla_3556	N/A	HBP
Sfla_3613	COG4188	dienelactone hydrolase
Sfla_3767	cd06260	domain of unknown function
Sfla_3776	N/A	hypothetical
Sfla_3819	N/A	hypothetical
Sfla_3854	N/A	HBP
Sfla_3855	pfam04149	BldB family
Sfla_3958	N/A	hypothetical
Sfla_4039	N/A	hypothetical
Sfla_4041	N/A	regulatory protein
Sfla_4131	pfam04149	BldB family
Sfla_4132	pfam01381	HBP
Sfla_4158	N/A	hypothetical
Sfla_4267	N/A	hypothetical
Sfla_4283	pfam00535	glycosyl transferase family 2
Sfla_4288	cd03786	UDP-N-Acetylglucosamine (GlcNAc) 2-Epimerase
Sfla_4402	N/A	Fasciclin-like protein
Sfla_4439	pfam04149	BldB family
Sfla_4440	N/A	HBP
Sfla_4606	pfam04149	BldB family
Sfla_4607	N/A	HBP
Sfla_4660	COG0841	Cation/multidrug effluN/A pump
Sfla_5089	COG0641	Arylsulfatase regulator
Sfla_5090	N/A	hypothetical
Sfla_5091	pfam00931	NB-ARC
Sfla_5365	pfam02618	YceG-like family
Sfla_5366	PRK00258	shikimate 5-dehydrogenase
Sfla_6367	N/A	hypothetical
Sfla_6381	pfam00440	tetR family regulator
Sfla_6389	N/A	hypothetical

A total of 59 core genes were found to have unusually high sequence variability (>5% nucleotide dissimilarity) between strains. These genes are listed using the IAF-45-CD sequence annotation and classified using CDD and RPSBLAST.

**Figure 5:**
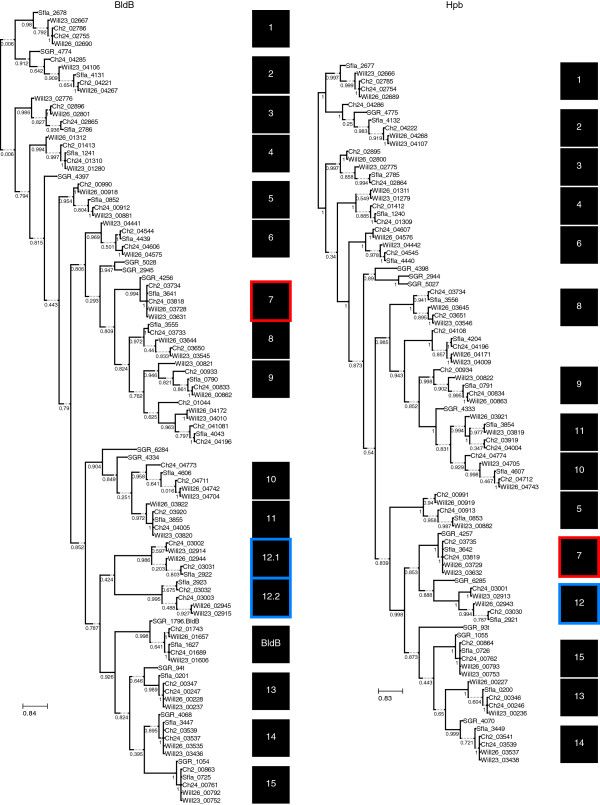
**BldB and Hpb phylogenies.** Maximum likelihood trees constructed from amino acid sequences of BldB homologs (left) and Hpb homologs (right). Sequence names indicate strain identity via locus tags. Values on edges represent SH-based local support values. The branch length represents evolutionary distance using the Gamma20 model within FastTree 2 [38]. Additional length is given to some branches with dashed lines to allow room for bootstrap values. Adjacent *bldB* and *hpb* genes are indicated in the two trees by numbered boxes to the right of the trees. Not all occurrences of *bldB* or *hpb* are accompanied by the other gene, as is the case with *bldB* originally characterized from *Streptomyces griseus* NBRC 13350 (SGR_1796.BldB). Box 7, highlighted in red, indicates incongruence between *bldB* and corresponding *hpb* gene phylogeny suggesting horizontal acquisition or gene conversion at one of these loci. Boxes 12.1 and 12.2, highlighted in blue, show an instance where *bldB* was duplicated locally without accompanying duplication of *hpb.*

**Figure 6:**
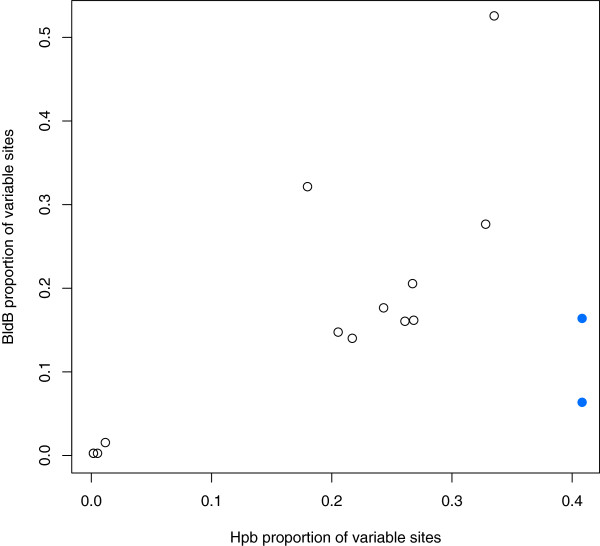
**Evidence for the co-evolution of BldB and Hpb pairs.** The proportion of variable sites in sets of orthologous BldB amino acid sequences is plotted against the Hpb sequences from the same locus. For example, there are 12 variable sites in the 76 amino-acid alignment of BldB sequences from group 1 in Figure 5, while the corresponding 274 amino-acid alignment for Hpb group 1 has 71 variable sites. The presence of amino acid variability in BldB is positively correlated with variability in Hpb at the same locus (Spearman’s ρ = 0.609, *p*-value = 0.01605). Points for the locus that corresponds to the internally duplicated *bldB* genes are shown in blue.

A total of 19 of the 59 divergent genes described above were found to belong to one of two gene families. One of these is putatively the transcriptional regulator *bldB*. Each genome contained 18 genes with homology to *bldB*. Nucleotide and amino acid alignments of *bldB* orthologs have a mean proportion of 0.118 (s.d. = 0.121) and 0.155 (s.d. = 0.142) variable sites, respectively. The other transcriptional regulator, which we refer to as helix-turn-helix partner of BldB (*hpb*), contains an N-terminal helix-turn-helix motif, but is otherwise as yet uncharacterized. Each genome contains 16 *hpb* homologs, and nucleotide and amino acid alignments of *hpb* orthologs have a mean proportion of 0.188 (s.d. = 0.126) and 0.208 (s.d. = 0.143) variable sites, respectively. We observe that genes of these families are typically adjacent or occur together as an operon. Only 2 of the 18 *bldB* homologs lack an *hpb* partner and only 1 of the 16 *hpb* homologs lacked a *bldB* partner. One putative operon (Sfla_2921-2923) contains two copies of *bldB* and only one copy of *hpb* (highlighted in blue in Figure 5 and Figure 6). The gene phylogenies are consistent with a general pattern of co-evolution with new gene pairs arising due to duplication, although lateral gene transfer has clearly affected the phylogeny (Figure 5). There is also a consistent relationship between the frequency of substitutions in the BldB orthologs and their Hpb partners (Figure 6).

### Effect of homologous recombination

SNP analysis revealed widespread recombination across the genome. Informative sites, which are sites at which two genomes share a SNP (*i.e.* it is not present in only one genome), can be evaluated with the four-gamete test [44] to identify polymorphisms acquired through recombination. The mean distance between incompatible sites is 1,191 bases indicating that each genome is a mosaic of intraspecies recombination. Informative SNPs shared by two strains support all possible patterns of ancestry among these five strains (Table 4) and this indicates that frequent recombination has erased the intraspecies phylogenetic signal across the genome. If this population were clonal, then informative SNPs would support a bifurcating pattern of ancestry with informative SNPs present only in strains that share a unique common ancestor to the exclusion of other genomes in the analysis.

**Table 4:** **Analysis of informative SNPs and variable genes**

	Sfla	Will23	Will26	Ch2	Ch24
**Sfla**	-	153	187	149	148
**Will23**	3334	-	303	323	361
**Will26**	4467	2947	-	291	274
**Ch2**	3865	2510	2538	-	360
**Ch24**	3367	2773	3317	2182	-

Pairwise analysis of informative SNPs are shown below the diagonal and shared variable genes present in only two genomes are shown above.

**Figure 7:**
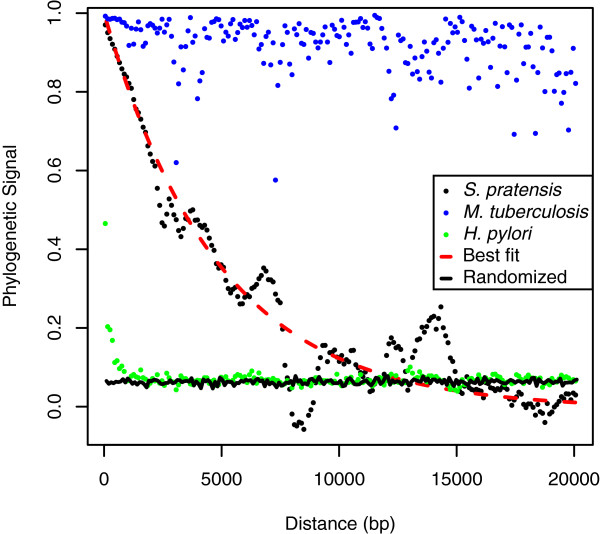
**Recombination erases the intraspecies phylogenetic signal in** ***S. pratensis.*** The proportion of incompatible sites along the five *S. pratensis* chromosomes is a function of chromosome distance. Phylogenetic signal is defined by a value of 1 indicating an absence of incompatible sites (pure vertical pattern of inheritance) and a value of 0 indicating that incompatible sites are randomly distributed by recombination. The solid black line indicates a random equilibrium SNP distribution generated from shuffling all SNPs among the *S. pratensis* genomes. Results are shown for the analysis of five *S. pratensis* genomes (black circles), as well as five *Mycobacterium tuberculosis* genomes which are known to represent a highly clonal species (blue circles) and five *Helicobacter pylori* genomes which are known to represent a highly recombining population (green circles). An exponential decay is fitted to the *S. pratensis* decay in phylogenetic signal due to distance between sites (dashed red line).

To determine the effect of recombination on phylogenetic reconstruction within this species, we determined the “phylogenetic signal” across the genome; a value of 1 indicates clonal inheritance, 0 indicates a random assortment of sites due to homologous recombination. For every informative SNP, the distance and compatibility of every other informative SNPs within 20 kb is used to calculate phylogenetic signal after combining the values into 100 bp bins. The phylogenetic signal in the *S. pratensis* genomes declined exponentially with distance (Figure 7). The nonlinear regression model fit to the data (implemented in R) is *y* = *e*^(-0.0002071*x*)^ where *y* is the phylogenetic signal and *x* is chromosomal distance. For comparison, the same analysis was performed on five genomes from the East Asian population of *Helicobacter pylori* (Figure 7), which is known to be highly recombinogenic [45]. Likewise, five genomes of *Mycobacterium tuberculosis,* a clonal species with low rates of recombination [46], were similarly analyzed (Figure 7). This result shows that the phylogeny supported by an informative site within *S. pratensis* has no bearing on the phylogeny of a site ≥15-20 kb distant.

**Figure 8:**
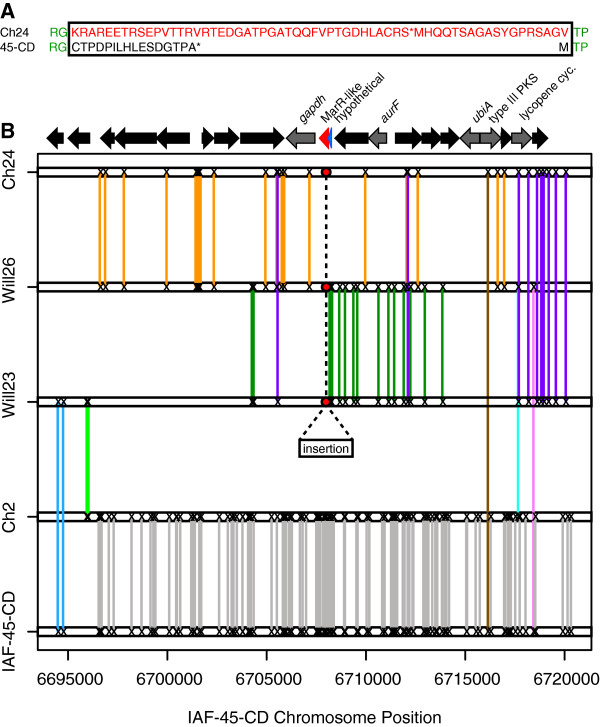
**Linkage region.** The *S. pratensis* genomes contain a 20 kbp region in which linkage equilibrium has broken down, suggesting a decrease in recombination between the genomes of Ch24, Will26, Will23 and those of Ch2, IAF-45-CD. The predicted amino acid sequences affected by an indel at the center of this region is shown in panel **A**. The amino acid sequences of Will26 and Will23 are identical in this region and differ from Ch24 by only one residue (only Ch24 is shown in panel A).The affected amino acid sequence is surrounded by a box. The location of the indel is indicated by a rectangle and red dots in panel **B**. Informative SNPs are indicated on each genome segment by an ‘X’ and colored lines indicate pairs of genomes which share each SNP. Stop codons are indicated with an asterisk in panel **A**.

The recombination analysis revealed an anomalous region of the genome that lacked incompatible sites over a span of 20,134 bp. The length of this region is unlikely to occur due to chance (*p* <0.01) based on the background rate of intraspecies recombination in the genome (Figure 7). This region was centered on an indel affecting genes Sfla_5857 and Sfla_5858 (Figure 8). These genes are annotated as a MarR-like regulator and a hypothetical protein, respectively. The insertion affects the terminal end of Sfla_5858 and the start of Sfla_5857. Also included in this region is a natural product biosynthetic gene cluster which comprises a type III polyketide synthase, a lycopene cyclase, a prenyltransferase (UbiA) and an AurF-like N-oxygenase (Figure 8). AurF is responsible for formation of the nitro- group in aureothin biosynthesis [47]. The indel mutation discriminates Ch24, Will26, and Will23 from Ch2 and IAF-45-CD (Figure 8). Linkage equilibrium has broken down around this indel with local patterns of recombination between genomes defined by the presence or absence of the indel mutation. The cause of linkage disequilibrium is unknown and could result from either a partial selective sweep enabled by horizontal transfer of this genomic island, selection against recombinants driven by the presence of the indel mutation in this region of the genome, or some unknown molecular mechanism which may be inhibiting recombination locally in response to the indel mutation.

A second genomic island is also found to lack incompatible sites, and the size of this region is unexpected to result from chance (*p* <0.01) based on the background rate of intraspecies recombination in the genome (Figure 7). The region occurs within a hybrid NRPS-PKS biosynthetic gene cluster (Sfla_6220-1) and spans 15696 bp. In this region the genomes IAF-45-CD and Ch2 do not have any evidence of recombination with the genomes Will23, Will26 and Ch24. This is the same pattern that is seen in the region of Sfla_5857-8 as discussed previously. The IAF-45-CD genome contains a single base insertion within this region that introduces a stop codon in a ketosynthase domain.

## Discussion

The size of the core genome can vary widely between bacterial species. For example, core genes can represent anywhere from 20% to 93% of the total genome across diverse bacterial lineages such as: *Escherichia coli*, ~20% [48]; *Streptococcus pneumoniae*, 52% [49]; *Salmonella enterica*, 61% [50]; *Actinobacillus pleuropneumoniae*, 79% [51]; *Listeria monocytogenes*, ~80% [52]; *Campylobacter coli*, 82.4% [53]; *Campylobacter jejuni*, 83.5% [53]; and *Chlamydia trachomatis*, 93% [54]. We have used a very strict definition of a core genome in our analysis of *S. pratensis*, using only positionally orthologous genes, as opposed to gene families, unlike many of the studies listed. Even with this strict definition, we calculate that 85-88% of each genome is comprised of core genes. Because our metric is more stringent than bi-directional best BLAST hits, which is used in many publications, these estimates should be viewed as a conservative estimate in comparison. Since the vast majority of variable genes are unique to a single genome (Figures 1A) and the estimate of core genome size changed little between the second and fifth genomes added to the analysis (Figure 1B) it is unlikely that the addition of new genomes will reduce substantially the size of the core genome in *S. pratensis*. Among published bacterial genome comparisons, only *Chlamydia trachomatis*, an obligate intracellular pathogen that has experienced extreme genome reduction and is left with only ~900 genes in the average genome [54], has a larger core genome proportion than *S. pratensis*. In addition, our assessment of the core genome does not exclude genes of putative plasmid origin, and as such represents a lower bound of core genome content for the linear chromosome. These observations suggest that the genome of *S. pratensis* is overwhelmingly dominated by core genes.

Comparative genomics of different species of *Streptomyces* has indicated that the central portion of the chromosome is highly conserved both in gene content and synteny while chromosome termini are highly variable between species. Our intraspecies comparison reveals islands of diversity spread throughout the chromosome, including at the terminal variable regions but also throughout the central core. The terminal variable regions are only 112 kbp (the 5’ arm as annotated in ATCC 33331) and 36 kbp (Figure 1D). This is significantly smaller than the 753 to 1,393 kbp terminal arms found in interspecific comparisons [55]. Strain specific islands are composed primarily of mobile genetic elements and genes that are likely to have been acquired by horizontal gene transfer. Strain specific islands composed of mobile genetic elements are a common feature of many microbial genomes.

All five of these genomes have the same repertoire of natural product gene clusters which are unambiguously part of the core genome of *S. pratensis*. This means that efforts to mine genomes for novel gene clusters will be facilitated by accurate species classifications, which can eliminate the need to needlessly screen many strains of the same species. However, our results for the *bldB* and *hpb* gene families suggest that changes to regulatory genes can occur at a fast rate within a species and may affect the expression of gene clusters between strains of a species. Examining diverse isolates from the same species may allow researchers to find regulatory changes that activate gene clusters that are otherwise cryptic in other strains. We have also shown that lanthipeptide precursors and NRPS and PKS genes can be highly variable within a species.

*S. pratensis* possesses a new combination of putative beta-lactam (MM 4550-like) and putative beta-lactamase inhibitor (clavulanic acid-like) biosynthetic gene clusters. This observation, on one level, suggests that observing patterns of natural product gene-clusters within the genomes of actinomycetes may reveal new possible drug combinations that have been proven effective by the crucible of evolution, and such new combinations may prove useful therapeutically. This observation also provides evidence for the *in situ* use of these products as antibiotics rather than as signaling molecules [56]. While the production of these molecules in *S. pratensis* has not yet been verified experimentally, the observation that convergent evolution has produced divergent combinations of beta-lactam and beta-lactamase inhibitor gene clusters independently in both *S. pratensis* and *S. clavuligerus* (species that do not share a common ancestor within *Streptomyces*) is evidence for the presence of an evolutionary arms race in soil communities. That is, selection has on at least two occasions driven the independent assembly of systems designed to produce both beta-lactam antibiotics and overcome beta-lactam resistance. The selection pressure for this pattern of gene cluster co-occurrence can only be explained by the hypothesis that: i) there is a high frequency of beta-lactam resistance in soils, and ii) these streptomyces benefit from the use of beta-lactam antibiotics to inhibit or kill other microorganisms in the soil community.

We have found that there is no consensus phylogenetic signal among *S. pratensis* genomes; the genome represents a mosaic of recombination between strains of the species. In this way *S. pratensis* resembles the East Asian *H. pylori* population in that recombination scrambles patterns of polymorphism between strains. A difference between these two species is that phylogenetic signal decays over shorter distances in *H. pylori* than in *S. pratensis* (Figure 7). This result could be caused by differences both in recombination rate and in the tract length of recombination. For example, *H. pylori* is naturally competent and can incorporate short stretches of DNA into its genome through transformation [45]. In contrast, acquisition of DNA by *Streptomyces* proceeds through an unusual dsDNA dependent mechanism of conjugation that may result in transfer of the whole chromosomes and backcrossing with the parent [7].

The frequency of recombination in *S. pratensis* was such that there is no detectable association between geographic distance and genomic divergence between strains from NY and NC. The geographic range of *S. pratensis* is unknown and it is not possible to estimate rates of migration from our current sample size, or to estimate whether dispersal limitation impacts the biogeographic pattern of genetic diversity within the species at very large spatial scales (e.g. continental). What we have shown, however, is that the extent of recombination between stains isolated from soils 740 km apart is not discernably different from the recombination observed between strains that co-occur in the same soil sample. Such a pattern could result from contemporaneous gene exchange at regional spatial scales (i.e. recombination between sites exceeds the ability of mutation to promote divergence between sites). Alternatively, this pattern could also result from the evolutionary recent regional expansion of a population that was recently in equilibrium.

While the majority of positionally orthologous core genes were highly similar between genomes, we observed 59 positional orthologs that differed by more than 5% between the *S. pratensis* genomes. Of these, 19 are members of two gene families known to be involved in regulation of differentiation and development. These two co-occurring gene families are BldB and what we have termed Hpb (for helix-turn-helix partner of BldB). BldB is a transcriptional regulator previously shown to be required for development of aerial mycelia, *e.g. bldB* mutants have bald colonies [57]. The conserved domain found within BldB is in the conserved domain database as DUF397 [34]. OrfD (SCO0703) in the antibiotic regulatory locus *abaA* and the developmental regulator WhiJ also fall within this class [58]. BldB has been characterized as a DNA-binding repressor that down-regulates its own expression [59], and was also previously suggested to have a binding partner that modulates BldB activity [60]. Hpb is predicted to have a helix-turn-helix domain of the Xre class. Based on the presence of a Xre domain, a history of gene duplication and recent proliferation, and limitation to a subset of the *Actinobacteria,* this gene pair was predicted by Makarova *et al*. [61] to represent a novel toxin-antitoxin system. The correspondence in diversification of the *bldb/hpb* family gene pairs suggests that a mutation in one gene encourages a compensatory change in its partner at the same locus. These observations from *S. pratensis* provide further indirect evidence that BldB-Hpb functions in a manner that resembles a toxin-antitoxin system.

## Conclusion

We have uncovered patterns of genome evolution within a *Streptomyces* species through comparison of *S. pratensis* genomes isolated from disparate geographic origins. Core genes make up a high percentage of the genome, and natural product genes are unambiguously included within the set of core genes. High rates of intraspecies recombination homogenize polymorphisms in core genes across the genome and abolish any phylogenetic signal present within species. Two genomic islands exhibited a breakdown in intraspecies recombination promoting localized divergence between sets of genomes. One of these islands was centered on a hybrid NRPS-PKS gene, and the other was centered on a regulatory gene associated with a type III polyketide synthase biosynthetic gene cluster. In addition, highly divergent core genes included a lanthipeptide biosynthetic gene cluster as well as co-occurring members of the *bldB* and *hpb* gene families, genes which have been associated with the regulation of development and antibiotic production in *Streptomyces*. The conservation of biosynthetic gene clusters between strains of the species suggests that antibiotic production capacity is conserved within a species while variation in *bldB* and *hpb* gene families suggest that changes to regulatory genes can occur at a fast rate within a species and may affect the expression of biosynthetic gene clusters between strains of a species.
